# Are we ready to be techno-digitalicus psychiatrists?

**DOI:** 10.1192/j.eurpsy.2023.194

**Published:** 2023-07-19

**Authors:** L. Orsolini

**Affiliations:** Unit of Clinical Psychiatry, Department of Neurosciences/DIMSC, Polytechnic University of Marche, Ancona, Italy

## Abstract

**Abstract:**

Digital mental health interventions and digital psychiatry have been rapidly implemented over the past decade, particularly with the intent to offer a potential solution in those problematic circumstances and logistic issues for which the current mental health service infrastructure is not able to adequately accommodate to the needs of most patients. Indeed, most mental health workforce does not always own an enough and appropriate theoretical either practical training in digital psychiatry and in delivering remote consultations safely and effectively. Most European countries do not have curricula-specific training requirements, either at core or higher specialty level, for psychiatry trainees to demonstrate competence in digital skills that may be considered essential to good clinical practice, including abilities and competencies needed to provide and deliver mental health interventions by using digital tools. The talk will provide an overview on the level of the current state-of-the-art of the European techno-digitalicus psychiatrist and psychiatry trainee.

**Disclosure of Interest:**

None Declared

